# Education and work participation among adults with congenital unilateral upper limb deficiency in Norway: A cross-sectional study

**DOI:** 10.1371/journal.pone.0207846

**Published:** 2018-12-12

**Authors:** Heidi Johansen, Trine Bathen, Liv Øinæs Andersen, Svend Rand-Hendriksen, Kristin Østlie

**Affiliations:** 1 TRS, National Resource Centre for Rare Disorders, Sunnaas Rehabilitation Hospital, Nesodden, Norway; 2 Innlandet Hospital Trust, Department of Physical Medicine and Rehabilitation, Ottestad, Norway; Washington University in Saint Louis School of Medicine, UNITED STATES

## Abstract

**Objectives:**

To describe level of education and work participation among adults with congenital unilateral upper limb deficiency (CUULD) in Norway and to explore associations between work participation and demographic and clinical factors.

**Methods:**

Cross-sectional study. In 2012, a postal questionnaire was sent to 186 persons with congenital limb deficiency (CLD), age ≥ 20 years, registered at the TRS National Resource Center for Rare Disorders. In the original CLD study, 77 persons with CUULD responded. In this paper 64 persons with CUULD of working age (20–67 years) are included. Data on demographic factors as education level and work participation, and clinical factors including limb deficiency characteristics, chronic pain (Standardized Nordic Questionnaire), fatigue (Fatigue Severity Scale), physical and mental health (SF-36) were analyzed through descriptive and comparable statistics and logistic regression analyses.

**Results:**

Sixty-four persons participated, mean age 43.4 (SD 13.7; range 20–67 years), 45 were women. Education level >13 years was reported by 34. Forty- three of the 64 participants were employed, 21 were prematurely retired (disability benefits). 11 of the 43 employed, and 6 of the 21 prematurely retired had completed vocational education. Physically demanding occupations (work activities that required standing, walking and lifting) were reported by 25 of the 43 employed and 13 of the 21 prematurely retired. 17 of the 64 reported need for further adaptions in their workplaces. The strongest predictors of work participation were younger age (OR 0.86) and good physical health (OR 1.21).

**Conclusion:**

Two thirds of persons with CUULD were employed; while one third was prematurely retired and had left work earlier than expected. This suggests that persons with CUULD may experience challenges in work participation. Although levels of education were relatively high, several had chosen careers that required physical strain. Younger age and good physical health were the most important factors mediating work participation.

## Introduction

This paper presents data on education (level and type) and work participation in adults with congenital unilateral upper limb deficiency (CUULD) in Norway. For adults, work is important to ensure personal development, financial security and social participation. There is however, limited research on education and work participation amongst adults with congenital limb deficiency (CLD) [1, 2, 3]. The Norwegian Limb Deficiency Association and proffesionals asked for more knowledge on these issues.

Deficiency of one upper limb is the most frequent CLD, and is twice as common as lower limb deficiency [4, 5]. CLDs are divided into transverse and longitudinal defects following the ISO/ISPO classification [6]. In transverse CLDs all structures distal to a specific point of the limb are lacking. In longitudinal CLDs, a bone or several bones are partially or completely lacking parallel to the long axis of the limb. Two hands are typically used in most daily activities and in work, one to grip and handle, the other for help and support. Persons with transverse upper limb deficiencies often totally lack grip in the deficiency arm. Persons with longitudinal deficiencies often have some kind of reduced grip [7]. Therefore, having CUULD causes early development of different, eventually automated, compensatory strategies [7]. Such strategies may be physically demanding over time.Some persons with CUULD may benefit from the use of prostheses; made for improving function, or for cosmetics purposes [8–10]. In Norway, rehabilitation, supply, procurement and maintenance of prostheses is free of charge for the prosthesis user, most often supplied by one of five limb deficiency centers [11].

The School system in Norway is tasked with giving students vocational guidance in order to enable paid work. The level of education achieved by individuals is an important factor for work participation [12]. In Norway most students go to upper secondary school after completing 10 years of compulsory basic education, choosing either vocational education (e.g. nurses assistant, carpenter, electrician) or general academic courses (preparation for college/university). Students finishing education after upper secondary school (13 years) will have access only to unskilled or vocational employment opportunities; low wage jobs, often with high levels of physical strain and few opportunities for adaption and variety. Students finishing higher education; bachelor degrees (16 years) or master/doctor degrees (>16 years) have a greater opportunity to choose less physically demanding work. When it comes to work, persons aged 20–67 are expected to work or study, and apporoximately 75% are in paid work [12]. Factors affecting work participation in the general population are complex. Fewer women than men participate in paid work [12] and research shows that low levels of education [12], chronic musculoskeletal pain [13], fatigue [14] and reduced physical or mental health may have impact on work participation [15].

We have only found three studies describing education and work participation in persons with CUULD [1–3]. The level of work participation appears to be high. Sjöberg et al. [1] found that 77% of persons with CUULD were employed in Sweden (mean age 33 years) and Postema et al. [2] found that 74% were employed in the Netherlands (mean age 41years). However, in a qualitative study, Lankhorst et al. [3] (also from the Netherlands) found that young adults with transversal upper limb reduction deficiency experienced limitations in finding suitable education and jobs because teachers and potential employers had doubts about their work ability. To our knowledge, only one study investigates factors predicting work participation in a combined group of persons with CUULD and acquired UULD [2]. Good physical health, prosthesis use, high education level and being a younger male were found to be predictors for work participation [2]. However, there seems to be a need for more knowledge on factors predicting work participation for persons with CUULD.

In an earlier publication, we reported a greater percentage of persons with CUULD on premature retirement (disability benefits) than in the Norwegian general population [11]. Studies have shown that individuals with CUULD are at risk of developing musculoskeletal pain [16–18], severe fatigue [16] and diminished physical health [18]. The aims of this paper therefore were to: 1) describe the level of education and work participation among adults with CUULD in Norway, 2) explore associations between work participation and demographic factors; age, gender and education level, and clinical factors; limb deficiency characteristics, comorbidities, pain, fatigue, physical and mental health.

## Patients and methods

### Design, subjects and ethics

**Fig 1:**
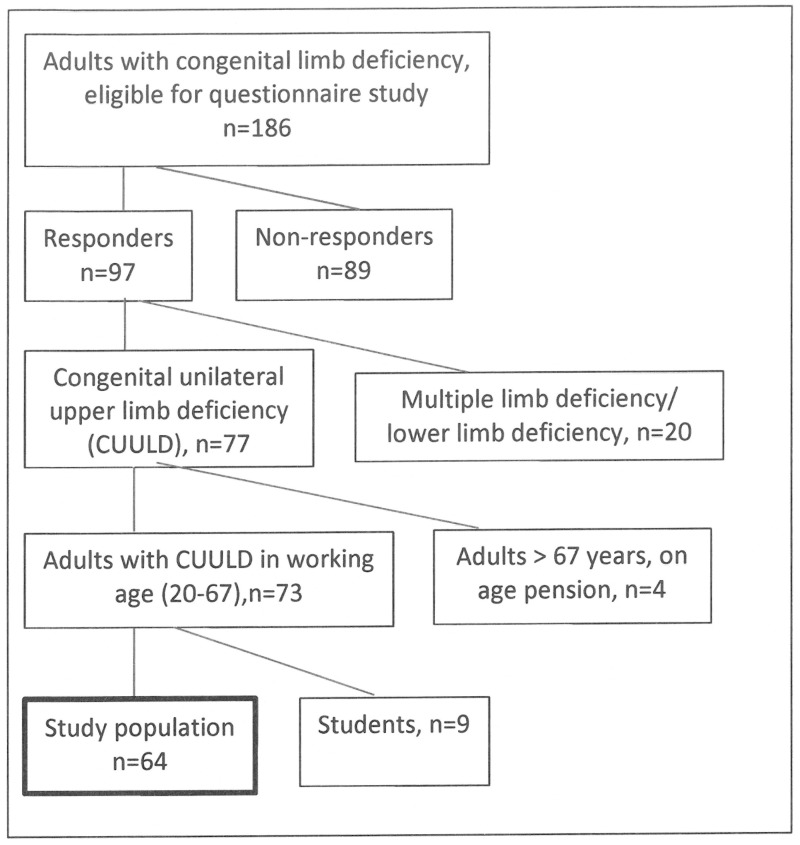
Inclusion of the participants.

In this paper we used data on individuals with CUULD of working age (20–67) (n = 64) from a cross-sectional, questionnaire-based study of adults with CLD [11, 16, 19]. Persons studying full time (n = 9) were excluded, because we assume that they have not yet any relevant experience with career based employment. Fig 1 shows the inclusion process for this paper.

In the original CLD study, individuals aged 20 or over with CLD, registered at the TRS National Resource Centre for Rare Disorders (TRS) in Norway were invited to participate. The exclusion criteria were lack of mastery of written Norwegian, syndactyly, polydactyly, and Poland syndrome without CLD. In October 2012, an information letter, together with a form for informed consent, a questionnaire and a prepaid, return-addressed envelope was sent to 186 adults with CLD. A written reminder was sent to non-responders after three weeks.

The Norwegian Southeastern Regional Ethics Committee for Medical and Health Research Ethics approved the study (No. 2012/805), as did the Data Protection Officer at the Oslo University Hospital.

### Questionnaire

A study specific questionnaire was designed in cooperation with the Norwegian Limb Deficiency Association. This questionnaire was evaluated in a pilot study [11, 16, 19]. Variables included were demographic and clinical factors.

#### Demographic factors

Demographic factors were gender, age, level of education (≤/ > 13 years), living alone / with a partner, and having children.

Work participation was investigated with questions gathered from health and labor force surveys in Norway and Sweden [12, 1]. The survey collected data on: 1. present employment status (paid employment vs. premature retirement (disability benefits)), 2. percentage of employment and premature retirement, 3. age at withdrawal from work if relevant, 4. degree of physical strain in work, four categories: a) sedentary work, b) work that requires much walking and standing, c) work that requires much walking and lifting, d) heavy manual work), 5. work challenges and work adaptions.

#### Clinical factors

A description of the CUULD (side, type and level for the affected limb, including marking the deficiency on a figure) was used to classify the deficiencies as left / right, transverse / longitudinal, and create the two variables; grip ability (no grip / reduced grip or near to normal grip) and amputation level (trans-humeral / trans-radial / finger-hand / longitudinal deficiencies). The classification was done in accordance with Day 1991 [6].

Other self-reported clinical factors with answer options yes/no, were: 1. comorbidity (do you have other diagnoses i.e. asthma, diabetes, rheumatoid arthritis, anxiety, depression or other, please describe), 2. use of prostheses, three categories: a) active (myoelectric or body-powered), b) passive (cosmetic) prostheses, c) a combination of active and passive prostheses, 3. cold sensitivity (do you freeze easily on the affected limb?).

Pain was measured with one item from the Standardized Nordic Questionnaire (SNQ) [20]: Have you experienced chronic musculoskeletal pain lasting more than three months, during the last year? (yes/no).

Fatigue was measured using the Fatigue Severity Scale (FSS), a nine-item questionnaire developed to measure the impact of fatigue on daily functioning [21]. Each item is rated on a 7- point response scale, ranging from 1 (completely disagree) to 7 (completely agree). A mean score is calculated for each person, with a range of 1 to 7 [22]. Higher scores indicate higher levels of fatigue. The Norwegian translation of the FSS has been used on the Norwegian general population (NGP) [22]. FSS is also used for different chronic diseases [23], and has been found valid and reliable [21–23].

To measure physical and mental health, we used the MOS SF-36 version 2 [24]. The SF-36 consists of 36 items, converted to eight subscales, four mental- and four physical scales. Mean scores may be reported for each individual subscale and for the two sum scores Physical Component Score (PCS) and Mental Component Score (MCS). All subscales in SF-36 have 0–100 scale, where 100 is best health status score [24]. Health related quality of life in our CUULD sample is reported in detail in an earlier paper [19]. In the present paper, we used the PCS and the MCS to explore associations between work participation and physical- and mental health.

### Data analysis

Data were entered into a customized database and processed using the Statistical Package for the Social Sciences (SPSS) version 19.0. The significance level was set at p ≤ 0.05.

Assessment of the representativeness of the original CLD sample (n = 186) (Fig 1), comparing gender, age, and place of residence (region) is reported in detail in a previous paper [8]. Analyses on the CUULD sample comparing amputation level, education and work participation was not possible because we did not have permission to collect this information on non-responders.

Descriptive statistics were used to analyze demographic and clinical factors. The study-population was divided into two groups: 1. Employed (working full time or part-time, no disability benefits), 2. Prematurely retired (disability benefits, full or partly). To explore differences between employed and prematurely retired, and between women and men, independent samples t-test was used for continuous variables, and Fisher`s exact test for categorical variables.

Univariate and multivariate logistic regression analyses were used to explore associations between the dependent variable work participation (employed vs. prematurely retired), and demographic and clinical factors. Independent dichotome variables were (yes/no): gender (women), living with a partner, education level (> 13 years), comorbidity, grip ability, prosthesis use, cold sensitivity and chronic pain. Independent continuous variables were: Age, Fatigue (FSS), physical and mental health (SF-36 PCS and MCS). Age, gender and the variables that were significantly (p<0.05) associated with work participation in the univariate analyses were entered simultaneously into a multivariate logistic regression analysis. The strength of each association was expressed as an odds ratio (OR) with a 95% confidence interval (95% CI). To evaluate the usefulness of the regression model, we used the Cox and Snell R square and Nagelkerke R square values to indicate the variation in the dependent variable. The variables amputation level and parenthood would also have been relevant to include in further analyzes, but were unsuitable because of limited cases in each category.

## Results

### Demographic and clinical factors

Data from sixty-four persons of working age are presented. Forty-four (69%) reported transverse deficiency, and 41 (64%) reported their deficiency to be left-sided. Further information on clinical factors is shown in Table 1.

**Table 1:** Demographic and clinical factors in the study group.

Characteristics	Study population (n = 64)	^a^ Employed (n = 43)	^b^ Prematurely retired (n = 21)	p-value
Mean age (SD)	43.4(13.7)	37.8(11.9)	55.0(9.1)	<0.001*
Gender, woman, n(%)	45(70)	29(67)	16(76)	0.568
Education level >13 years, n(%)	34(53)	27(63)	7(33)	0.015*
Basic compulsory education (10 y), n(%)	3(4)	1(2)	2(10)	
Upper secondary school vocational education (13 y)n(%)	17(26)	11(26)	6(28)	
Upper secondary school general courses (13 y), n(%)	10(16)	4(9)	6(28)	
University/college (bachelor 16 y), n(%)	21(33)	16(37)	5(24)	
University/college (master/PhD etc. > 16 y), n(%)	13(20)	11(26)	2(10)	
Mean age (SD) receiving disability benefits		-	45.0(8.8)	
Living with partner, n(%)	46(72)	30(70)	16(76)	0.719
Parenthood, n(%)	48(75)	27(63)	21(100)	<0.001*
Amputation type and level				
Trans-humeral deficiency, n(%)	2(3)	2(5)	0	1.000
Trans- radial deficiency, n(%)	30(47)	18(42)	12(57)	0.294
Finger/hand deficiency, n(%)	12(19)	7(16)	5(24)	0.507
Longitudinal deficiency, n(%)	20(31)	16(37)	4(19)	0.164
^c^ Comorbidity n(%)	28(44)	14(33)	14(67)	0.013*
Grip ability in deficiency limb, n(%)	14(22)	11(26)	3(14)	0.356
^d^ Prosthesis user, n(%)	31(48)	19(44)	12(57)	0.427
Cold sensitivity, n(%)	44(69)	28(65)	16(76)	0.407
Chronic musculoskeletal pain, n(%)	42(66)	23(54)	19(91)	0.004*
Mean(SD) fatigue (FSS)	4.0(1.5)	3.8(1.5)	4.4(1.5)	0.126
Mean (SD) SF-36 Physical Component Summary	46.0(8.9)	49.3(8.2)	39.5(6.6)	<0.001*
Mean (SD) SF-36 Mental Component Summary	47.8(11.5)	48.9(10.0)	45.5(14.0)	0.322

^a^ Employees include persons who work full time (n = 33) or part time (n = 10) (without any disability benefit)

^b^ Prematurely retired (disability benefits) include persons on disability benefits 100% (n = 13), partial disability benefits (n = 8)

^c^ Asthma/Allergy, n = 14. Anxiety/ depression, n = 8. Metabolism high/ low, n = 4. Diabetes, n = 3. Psoriasis, n = 2.

Arterial fibrillation, n = 2. Blood pressure high/ low, n = 2. Ankylosing spondylitis, n = 1. Schizophrenic, n = 1. Liver disease, n = 2. (NB: a person may describe more than one comorbidity)

^d^ Prosthesis user is a person who uses an active (myoelectric or body powered) prosthesis, passive (cosmetic) prostheses, or a combination of the two.

* Statistics significant differences, p< 0.05.

**Fig 2:**
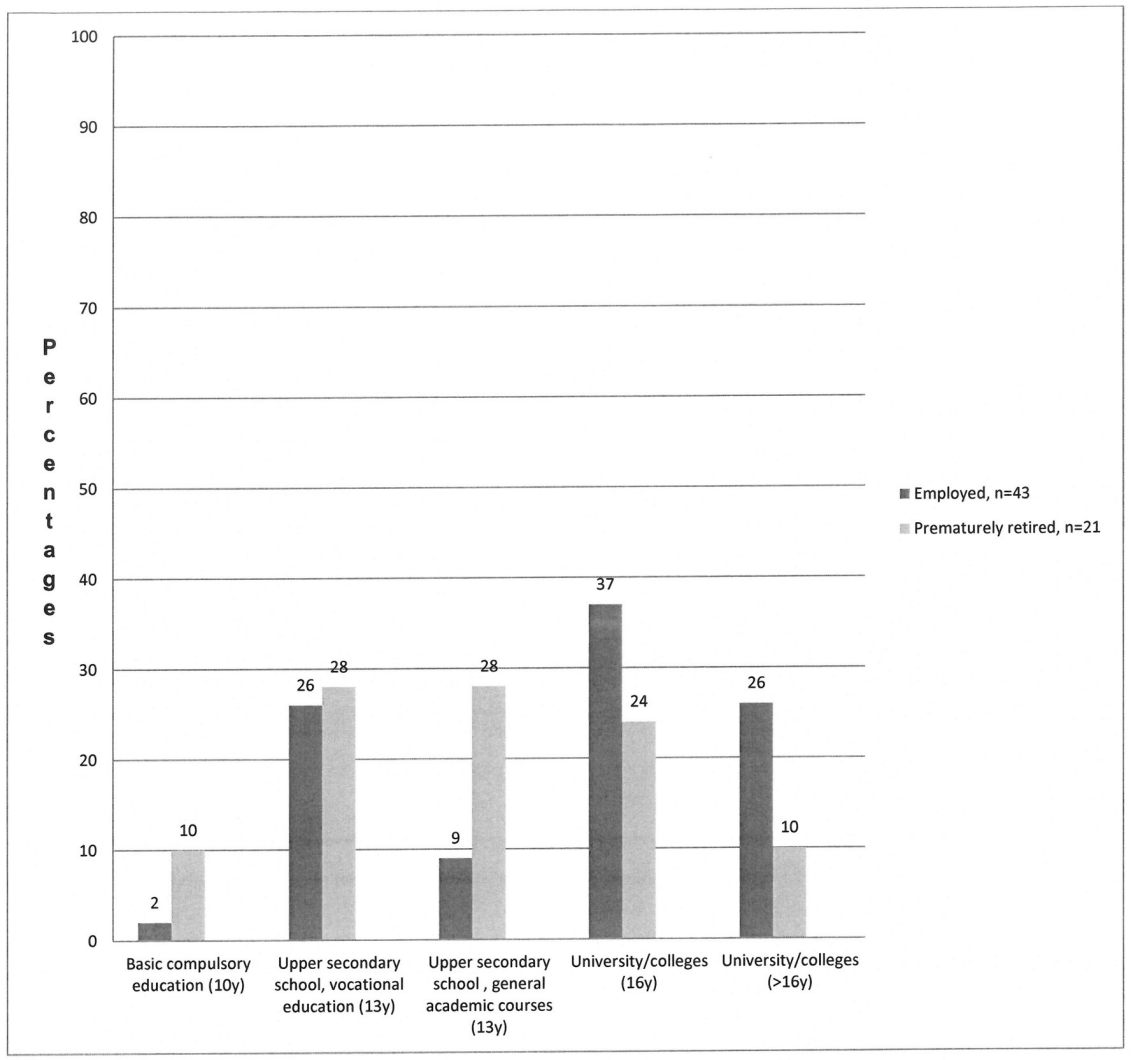
Percentages of employed persons and persons prematurely retired, dependent on education level.

The mean age for the study population was 43.4 years (SD 13.7, range 20–67 years), and 45 (70%) were women. Thirty-four (53%) reported an education level of more than 13 years (Table 1). The education levels attained for both employed and prematurely retired individuals are shown in Fig 2. Forty-three persons (67%) were employed and 21 (33%) were prematurely retired. The mean age (SD) for receiving premature retirement was 45.0 (8.8) years, (range 20–57). Significant differences were found between employees and prematurely retired persons concerning age, education level, parenthood, comorbidity, chronic pain and physical health (Table 1). No significant differences were found between women and men regarding premature retirement (p = 0.568) and age for leaving work (p = 0.661).

### Work strain and work adaptions

**Fig 3:**
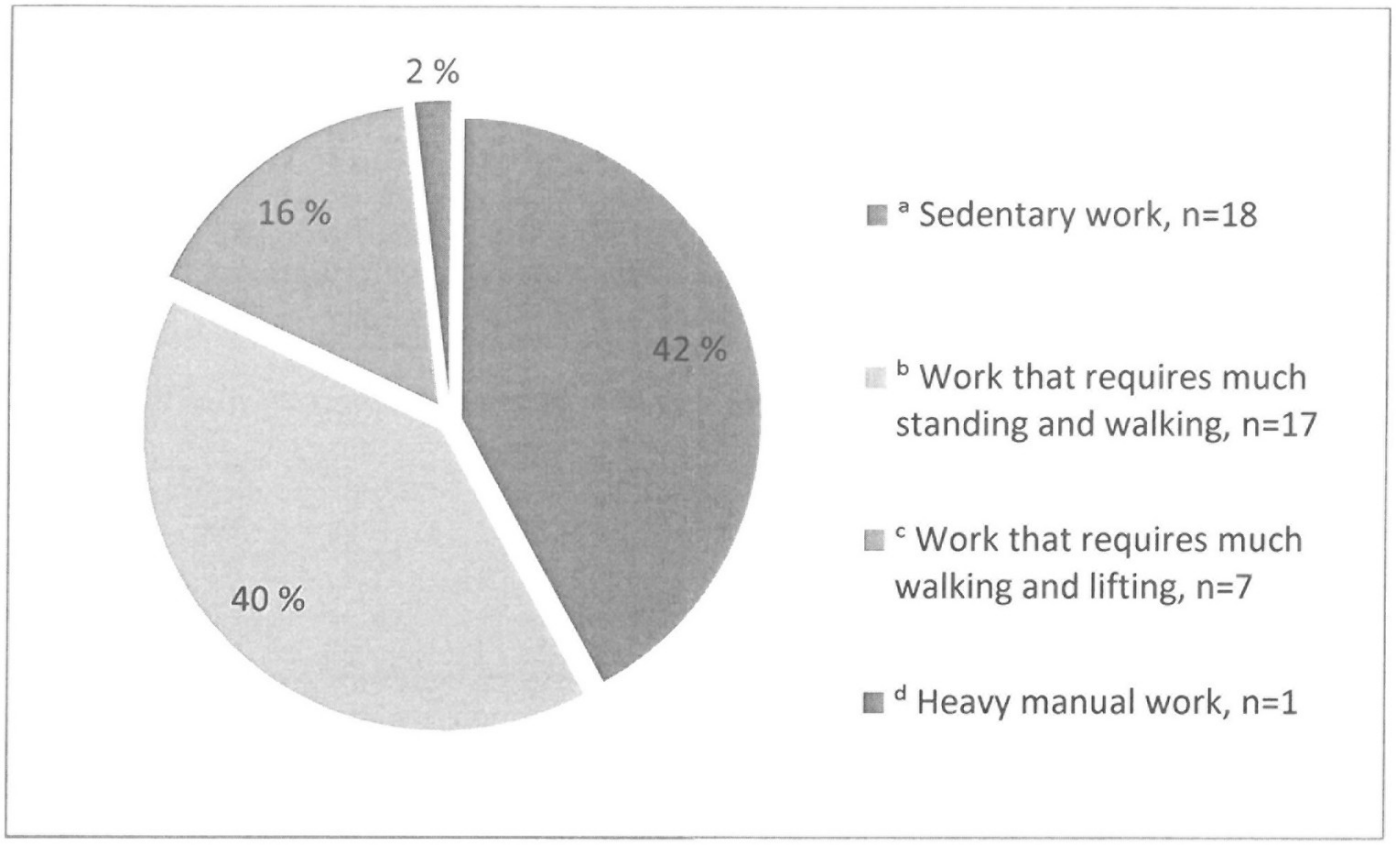
Degree of physical strain for employees. Working full time, n = 33, working part-time, n = 10. ^a^ Sedentary work: for example deskwork, administration; ^b^ Work that requires much standing and walking: for example shop assistant, light industry, teaching; ^c^ Work that requires much walking and lifting: for example postman, nurse, construction worker; ^d^ Heavy manual work: for example forest worker, heavy farming work, heavy construction work.

Fig 3 shows the degree of physical strain in work for employees. Most of the employed were in sedentary work, but a considerable proportion was in occupations that were physically demanding (requiring much standing and walking, or much walking and lifting). Of the prematurely retired 8 out of 21 individuals had been in sedentary work occupations, 7 of 21 in occupations that required standing and walking, and 6 of 21 in occupations that required walking and lifting.

Table 2 shows self-reported work challenges and work adaptions for both employees and prematurely retired individuals (for the latter: their experience when they still were employed). Compared to employees, significantly more retirees reported that pain had inhibited their work ability, that their limb deficiency had affected their choice of education and work, and that they had received vocational guidance. More employees reported having been met with understanding at work.

**Table 2:** Work challenges and work adaptions for employees and prematurely retired with CUULD, n = 64.

Challenges and work adaptions (Yes/no)	Employed n = 43	Prematurely retired n = 21	Differences (p-value)
Does/did pain inhibit your work ability? Yes, n(%)	6 (14)	7 (33)	0.041*
Did the limb deficiency affect your choice of education? Yes, n(%)	12 (28)	14 (66)	0.005*
Did the limb deficiency affect your choice of work? Yes, n(%)	13 (30)	15 (71)	0.002*
Are/was work situation adapted to the limb deficiency? Yes, n(%)	4 (9)	5 (24)	0.120
Does/did you need more work adaptions? Yes, n(%)	12 (28)	5 (24)	0.488
Are/was you met with understanding at work? Yes, n(%)	33 (77)	7 (33)	0.001*
Does/did the limb deficiency reduce your work capacity? Yes, n(%)	9 (21)	7 (33)	0.209
Did the limb deficiency influence your retirement? Yes, n(%)	-	15(71)	-
Have you received any vocational guidance? Yes, n(%)	6 (14)	8 (38)	0.033*

Fisher Exact Test

* Statistical significant differences, p< 0.05.

### Work participation and associated factors

Education level, chronic pain, comorbidity, age and physical health were significantly associated with work participation in the univariate analyses (Table 3). In the multivariate logistic regression model, work participation was associated with being younger (aOR = 0.86, p = 0.015) and with better physical health (higher PCS score) (aOR = 1.21, p = 0.024). This model explained 52.3% (Cox and Snell R square) and 72.4% (Nagelkerke R square) of the variance in work participation, respectively (Table 3).

**Table 3:** Associations between work participation (employed vs. prematurely retired) and demographic and clinical factors (n = 64).

Independent variables	Univariate logistic regression Crude effect estimates^a^			Multivariate logistic regression Adjusted effect estimates^b^		
	cOR	95%CI for cOR	*p* cOR	aOR	95%CI for aOR	*p* aOR
Gender, women						
Yes	0.65	0.20 to 2.13	0.474	NI		
No	Ref	Ref				
Living with a partner						
Yes	0.78	0.23 to 2.61	0.688	NI		
No	Ref	Ref				
Education > 13 years						
Yes	4.2	1.34 to 13.2	0.014*	4.63	0.35 to 61.12	0.244
No	Ref	Ref				
Comorbidity						
Yes	0.21	0.07 to 0.68	0.009*	0.28	0.04 to 1.89	0.189
No	Ref	Ref				
Prosthesis user						
Yes	0.59	0.21 to 1.72	0.332	NI		
No	Ref	Ref				
Grip ability						
Yes	2.12	0.51 to 8.37	0.311	NI		
No	Ref	Ref				
Sensitive to cold						
Yes	0.58	0.18 to 1.93	0.372	NI		
No	Ref	Ref				
Chronic pain						
Yes	0.12	0.03 to 0.59	0.009*	0.60	0.05 to 7.00	0.683
No	Ref	Ref				
^c^Age	0.87	0.82 to 0.93	<0.001*	0.86	0.77 to 0.97	0.015*
^c^Fatigue Severity Scale	0.76	0.53 to 1.12	0.126	NI		
^c^SF36 Physical component score	1.17	1.10 to 1.27	<0.001*	1.21	1.03 to 1.42	0.024*
^c^SF36 Mental component score	1.03	0.98 to 1.10	0.263	NI		

Abbreviations: cOR = crude Odds Ratio; aOR = adjusted Odds Ratio; CI = confidence interval; NI = not included in adjusted model; Ref = reference category.

^a^ Crude effect estimates: logistic regression analysis, one independent variable in the model at a time

^b^ Adjusted effect estimates: logistic regression analysis, estimates adjusted for the included covariates

Gender, women: yes = 1, no = 0. Living with partner: yes = 1, no = 0, Education level >13 years: yes = 1, no = 0. Chronic pain: yes = 1, no = 0. Comorbidity: yes = 1, no = 0. Grip ability: yes = 1, no = 0. Sensitive to cold: yes = 1, no = 0. Prosthesis user: yes = 1, no = 0.

^c^ Continuous variables: Age, Fatigue severity scale (1–7). SF-36 Physical component score (0–100). SF-36 Mental component score (0–100). This model explains 52.3 to 73.4% of the variability in work participation.

* Statistics significant differences, p< 0.05.

## Discussion

### Main findings

In this Norwegian study, most persons of working age with CUULD reported a high educational level. However, a quarter of the study population had chosen vocational education. About one third were prematurely retired with a mean age of 45 years. Among the employed approximately two thirds were employed in physically demanding work, i.e. work that required much standing, walking, lifting or heavy manual work. Younger persons and persons with high self-reported physical health score were most likely to be employed.

### Employment vs. early retirement

In this study 67% were employed. This is lower than the 75% reported in the Norwegian general population (NGP) [12], and than previously reported for persons with CUULD [1, 2]. A considerable proportion of our respondents received full time disability benefits (20%), higher than in the NGP (10%) [12]. The mean age for leaving work is also lower than expected in the NGP (45 vs. 67 years). This has not been previously reported among persons with CUULD. The mean age for leaving work may be influenced by differences in health and welfare systems in different countries. One possible explanation for the high proportion of prematurely retired may be that persons with apparent functional problems are likely to be believed and understood for their problems in the health and welfare service system. Thus, when they apply for disability pensions, they will most likely have these granted. Also, Norway has welfare programs that make it possible to end one`s career early for health reasons, while still living a decent life.

In the NGP, [12] more women than men were on disability pensions, and stopped working at an earlier age. We did not find such gender differences, which may be due to the gender distribution in the study population, with relatively few men.

In our CUULD sample a quarter of respondents had chosen careers that required physical strain, despite their physical challenges, in contrast with the findings of Postema et al. [2] yet in accordance with Sjöberg et al. [1]. Some may have chosen their education and employment to demonstrate that the lack of a hand should not prevent them from doing what they found important and meaningful. This attitude is also known from our clinic, and was found in an interview study among adult women with CUULD [25]. However, as for the general population, some persons with CUULD do not have the desire or capacity to fulfill higher education. They simply wish to finish their compulsory basic education and then move into paid work. Furthermore, as Postema et al. [2] point out careers involving primarily mental work can also be perceived as physically demanding for persons lacking one arm.

### Work challenges and work adaptions

Only 14% of the respondents reported that their work situation had been adapted to their limb deficiency, and 27% reported requiring further adaptions. This is in contrast to a study of Norwegian persons with different kind of disabilities, were 59% reported having adaptions in their work situation [12]. Our results indicate that many individuals do not have necessary adaptions of their work situation. However, we do not know whether such adaptions were not offered, or if the employees simply chose not to use them.

As Lankhorst et al. [3] pointed out, some persons with CUULD, wanting to appear like everyone else, may not seek advice from rehabilitation teams or professionals who know about their physical challenges. Our experience is that children with CUULD receive rehabilitation in one of Norway`s five limb deficiencies. In adolescence many drop out of routine follow up, and some seek rehabilitation when pain occurs later on in life. Targeted guidance and information on challenges and relevant adaptation options may influence at least some individuals with CUULD to choose education and careers that might allow them to stay in work longer.

### Work participations and associated factors

In the multivariate logistic regression analysis, only age and physical health were found to be significantly associated with work participation. Unsurprising, younger persons were most likely to be employed. This is in accordance with Postema et al. [2] who found that younger age was a predictor of work participation in persons with CUULD.

In line with Postema et al. [2] we also found that good physical health influenced the ability to remain in employment. This is also in line with our clinical experience. As Postema et al. and Lankhorst et al. also point out, strategies intended to preserve or improve physical health may be important for work participation [2, 3].

In contrast to the findings of Postema et al. [2] however, educational level was not significantly associated with work participation in our study. Further research on this matter is needed. Persons with CUULD in our study reported a high level of education, also compared to the NGP [26]. This is in accordance with other studies on CUULD [1, 2]. A high level of education is also found among other groups with congenital physical disabilities [27–29]. Howerver, Lankhorst et al. [3] found that more young adults with upper limb reduction deficiency had faced skepticism from teachers and potential employers in relation to career choice and work.

Grip ability, sensitivity to cold and prosthesis use was not associated with work participation in our sample. This is in line with Vasulian et al. [9] who found that youngsters with CUULD manage well with compensatory techniques developed throughout life, and that use of a prosthesis may in fact disturb their natural movement patterns and interfere with sensibility. In opposition to this, Postema et al. [2] found prosthesis-use important for work participation, and they suggest that not only the function, but also the cosmetic aspect of prosthesis-use may be important. It is likely that the cosmetic aspect is just as important in Norway as in the Netherlands and Sweden.

A high prevalence of chronic musculoskeletal pain has been reported in several studies on persons with congenital and acquired UULD [16–18]. Chronic pain is a predictor for work participation in the general population [13]. However, we did not find any association between chronic pain or fatigue and work participation in our sample. Postema et al. [2] also did not find that pain was associated with work participation; however, they did find that pain was related to work productivity. The impact of chronic musculoskeletal pain on work participation and productivity in persons with CUULD should therefore be elaborated upon in further studies.

Fatigue in adults with CUULD has not been investigated previously, and might be important in further studies. In a previous paper [16], we reported severe fatigue among one third of the study population. Furthermore 25% of respondents in the present study reported reduced work capacity. This is in line with Sjöberg et al. [1] who found that adults with congenital limb deficiency reported reduced general work capacity. It is likely that this to some extent reflects impact of the limb deficiency on an individual’s physical capacity which may become visible as mental strain and fatigue.

### Strength and weaknesses

A low response rate is a weakness and may have led to selection bias in our sample. As previously reported [11] the respondents in the original CLD study were older, and with a lower percentage of men than the non-responder group. This may have affected our findings regarding variables that are known to be gender-dependent, such as work participation [12]. As described in detail elsewhere [11, 16, 19] recruiting persons with CUULD only from the TRS National Resource Center for Rare Disorders in Norway may have resulted in selection bias. However, our sample may be more representative than samples recruited from specialized hospital clinics, as all persons with CUULD regardless of problems may use TRS services.

The use of self-reported data may give recall bias, and small sample size gives reduced statistical power for several analyses, especially regarding differences between subgroups. This implies that our results may not be generalized to the whole CUULD population.

## Conclusions

Two thirds of persons with CUULD were employed; while one third were prematurely retired and had left work earlier than expected. This implies that some individuals at least experience challenges in work participation. Although more than half of our sample had a high level of education, our findings indicate that a considerable proportion had chosen upper secondary school with vocational education. Many had careers that entailed physical strain, including standing, walking and lifting. We found that younger age and good physical health were the most important factors mediating work participation.

A greater focus on vocational guidance early in life, use of adaptions in work situations, and strategies to deal with reduced physical function might support persons with CUULD to manage their careers and hopefully delay early retirement.
